# Basket trials in rare diseases: a systematic review of current practices, methodological challenges, and future directions

**DOI:** 10.1186/s13023-025-04048-w

**Published:** 2025-11-12

**Authors:** Wael Khazen, Solange Corriol-Rohou, Teresinha Evangelista, Arnaud Valent, Sarah Abbas, Xavier Nissan, Alexandre Mejat

**Affiliations:** 1 AFM-Telethon, 1, rue de l’Internationale, BP 59, 91002 Evry cedex, France; 2 SCR Consulting, 75016 Paris, France; 3 Unité de Morphologie Neuromusculaire, Institut de Myologie, Paris, France; 4 Généthon, 1 bis, rue de l’Internationale, 91002 Evry cedex, France; 5 Université Paris-Saclay, Université d’Evry, Inserm, IStem, UMR861, 91100 Corbeil-Essonnes, France; 6 IStem, CECS, 91100 Corbeil-Essonnes, France

## Abstract

The development of therapies for rare diseases (RDs) continues to face persistent challenges, including small and geographically dispersed patient populations, pronounced clinical heterogeneity, and the absence of standardized outcome measures. Basket trials—master protocol studies evaluating a single therapeutic intervention across multiple diseases linked by shared molecular or clinical characteristics—offer a promising solution to these constraints. This systematic review identified 36 basket trials targeting RDs through comprehensive searches of clinical trial registries, academic databases, and grey literature. The majority (75%) focused on rare oncological indications, with only nine trials addressing non-oncological RDs. These non-oncological studies were highly heterogeneous, spread across 25 distinct conditions without overlap, and faced persistent challenges such as the lack of validated biomarkers and standardized endpoints. Most studies (81%) were Phase II trials, highlighting the exploratory role of basket designs in early-stage development. Trial designs were predominantly non-randomized and open-label (86%), reflecting the practical limitations of implementing rigorous methodologies in small, heterogeneous populations. The average trial duration was 6.5 years, and recruitment was logistically demanding, with trials involving a mean of 56 sites and, in some cases, over 1,000 centers. While basket trials show clear potential to accelerate therapeutic innovation in RDs, their application remains limited beyond oncology. Methodological constraints—such as inconsistent endpoints, limited randomization, and underpowered subgroup analyses—continue to restrict their broader use. Enhancing the utility of basket trials will require greater regulatory flexibility, wider adoption of adaptive and Bayesian designs, integration of real-world evidence, and stronger engagement with patients and advocacy groups. This review underscores both the opportunities and limitations of basket trials in RDs and provides a roadmap for realizing their potential, calling for concerted efforts from regulators, researchers, and patient advocates to expand their application and impact across the RDs spectrum.

**Supplementary Information:**

The online version contains supplementary material available at 10.1186/s13023-025-04048-w.

## Introduction

Rare diseases affect approximately 350 million people worldwide, with an estimated 30–40 million individuals in the European Union alone, corresponding to 6–8% of the global population [1, 2]. Despite their collective burden, most RDs remain without disease-specific therapies, and affected patients often face delayed diagnosis, limited treatment options, and poor access to specialized care. Approximately 80% of RDs have a genetic origin, with 50–75% manifesting in childhood, frequently leading to severe morbidity and high early mortality rates [3]. Despite increasing attention to RDs, approximately 95% still lack approved treatments, and nearly one-third of affected children do not survive beyond the age of five [3]. Among the few therapies that do exist, access remains limited—globally, fewer than 10% of patients benefit from disease-specific interventions. This reflects a combination of factors, including the absence of approved therapies for most RDs, restricted geographic availability of approved drugs, and, in some cases, the lack of positive reimbursement decisions despite marketing authorization [4]. Moreover, treatment costs pose a major barrier, with orphan drugs priced up to 13.8 times higher than conventional medicines [5, 6]. Although the U.S. Food & Drug Administration (FDA) has approved therapies for around 500 rare conditions, the vast majority of these remain unavailable to nearly 90% of patients globally, highlighting persistent disparities in access and affordability [7].

The clinical development of therapies for RDs continues to encounter significant, well-documented challenges. Patient recruitment is often complicated by the small size and wide geographical dispersion of patient populations, alongside a frequent lack of standardized treatment guidelines. In many protocols, eligibility criteria further require patients to be treatment‑naïve, which can significantly reduce the pool of potential participants. Disease heterogeneity, limited population numbers and lack of validated biomarkers further complicate trial design, endpoint selection, data interpretation, and ultimately the regulatory approval process. In addition, the variability in age of onset and, for some conditions, a slow and heterogeneous disease progression add further complexity, making it difficult to define appropriate inclusion criteria and to measure clinically meaningful changes within typical trial timeframes. In many instances, knowledge gaps persist regarding disease mechanisms, natural history, and clinically meaningful outcomes. These factors, coupled with the high costs of development and modest commercial incentives, have historically constrained investment in this field [5, 8, 9].

Addressing these barriers requires innovative trial designs that optimize patient inclusion and strengthen statistical power through adaptive methodologies, biomarker-guided enrolment, and decentralized approaches. The overarching goal is to enhance efficiency and clinical relevance, thereby enabling earlier access to effective therapies once regulatory and reimbursement hurdles are overcome. Among the strategies proposed, master protocol trials, a category that includes basket, umbrella, and platform designs. These frameworks allow simultaneous evaluation of multiple hypotheses within a single overarching trial. Basket trials—the focus of this review—test a single intervention across diseases, conditions, or subtypes sharing common biological features such as genetic mutations, biomarkers, histology, or age of onset [10]. In contrast, umbrella trials investigate multiple therapies in one disease, while platform trials permit ongoing modifications such as adding or dropping arms.

Advanced statistical approaches, including hierarchical and Bayesian modelling, can also leverage information across subgroups to increase precision while maintaining disease-specific analyses [11].

Unlike conventional RCTs, which face major recruitment barriers in rare diseases, basket trials provide a more flexible and pragmatic framework for targeted therapy evaluation. Master protocols have substantially advanced clinical research, especially in oncology, where basket trials enabled stratification by shared molecular biomarkers and accelerated the development of precision therapies. While most experience has been in cancer, applications are now emerging in other fields, including Alzheimer’s disease [12, 13], vasculitis [14], metabolic disorders [15], and infectious diseases [16].

Despite this progress, the use of basket trials in RDs research remains underutilized. Biological complexity, smaller sample sizes, and a lack of established biomarkers continue to pose significant methodological and operational challenges [17]. However, emerging evidence underscores their potential to reshape RD drug development by clustering patients with shared genetic mutations, regardless of clinical phenotype [18]_._ This paradigm shift aligns with the broader evolution of precision medicine, wherein therapies are increasingly tailored based on molecular pathophysiology rather than traditional disease classifications [19].

Although the use of basket trials in RDs remains limited, successful examples have begun to emerge. A notable case is the vemurafenib basket trial (VE-BASKET), which assessed the efficacy of vemurafenib, a kinase inhibitor, in patients with BRAF V600E mutation-positive Cancers. This trial demonstrated significant clinical benefits, particularly for individuals with Erdheim-Chester disease and Langerhans cell histiocytosis (LCH) [18]. A pivotal regulatory milestone occurred in 2017 when the U.S. FDA approved vemurafenib for patients with BRAF V600E mutation–positive cancers, irrespective of tumor histology. This landmark decision validated the concept of enrolling patients based on a shared molecular biomarker rather than disease type, setting an important precedent for biomarker-driven basket trials and regulatory pathways [20]. That same year, the FDA granted the first tissue-agnostic approval to pembrolizumab based on basket trial data, further underscoring the potential of this design to transform therapeutic development across both rare and more common diseases [21].

Data from the GlobalData database further highlight the increasing use of basket and umbrella trials in RDs. Between 2016 and 2021, the number of industry-sponsored basket trials in RDs increased, rising from approximately 10 to 40 [17]. However, despite the initiation of over 5,000 RDs clinical trials in 2021 alone [22], basket trials remain underused. This reflects both scientific opportunities and legitimate challenges, including regulatory uncertainties, difficulties in endpoint harmonization, powering subgroups, and the logistical complexity of running multi‑cohort studies. This gap highlights the importance of improving awareness and understanding of basket trial principles, including their methodological foundations, advantages, and limitations, to facilitate broader adoption in both exploratory and confirmatory trial settings.

Regulatory bodies have begun to recognize the value of innovative designs, issuing guidance and supporting their adoption through multi-stakeholder initiatives. The FDA and the European Medicines Agency (EMA) have played pivotal roles in this paradigm shift [23, 24]. In the same way, industry groups such as the European Federation of Pharmaceutical Industries and Associations (EFPIA) convened a multistakeholder workshop in 2021 to promote the use of complex trials across Europe and beyond [25].

The launch of the European Partnership on Rare Diseases (ERDERA) in October 2024 under the Horizon Europe program highlights the growing momentum behind collaborative efforts to advance RDs research through innovative trial designs and cross-sector cooperation [26, 27]. This commitment is echoed by several other major initiatives, including the Innovative Health Initiative (IHI) Comprehensive Methodological and operational Approach to Clinical Trials in Rare Diseases (Realise D) consortium [28], which is working to streamline and optimize the development of therapies for RDs. The Drug Repurposing and AI for Muscular Disorders (DREAMS) project, also funded under Horizon Europe, focuses on developing and validating cross-disease methodologies, particularly the use of patient-reported outcomes and innovative trial designs in neuromuscular diseases [29]. Other public–private partnerships, such as INVENTS [30], also contribute to this collective ambition by promoting methodological innovation and shared infrastructure.

To provide a comprehensive overview of the current landscape of basket trials in RDs, we conducted a systematic literature review of both completed and ongoing studies at the time of our search (25 February 2025). The primary aim was to examine how these trials have been designed and implemented, the methodological frameworks they employ, and the outcomes they report—while highlighting both their potential advantages and recurring limitations. Rather than addressing the statistical intricacies or evaluating the comparative robustness of trial designs, this review focuses on their conceptual foundations and practical relevance within the context of RDs research.

Through this review, we sought to characterize and describe the different types of basket trials conducted to date—whether in rare oncology or non-oncological RDs —with particular attention paid to trial design, methodological strategies, and patient recruitment processes.

In addition, we carried out a comparative analysis with oncology-based basket trials to highlight important distinctions, shared challenges, and transferable insights that could guide the future design of basket trials in non-oncological settings. By synthesizing these findings, our review offers strategic recommendations to enhance the use of basket trials in RDs —promoting more efficient, scalable, and inclusive approaches to therapeutic development.

## Methods

To comprehensively identify and analyze all ongoing and completed basket clinical trials in the field of RDs, we conducted a systematic search between February 17 and 21, 2025, adhering to the PRISMA guidelines for systematic reviews [31]. Our search targeted all relevant publications available up to February 21, 2025, and encompassed 2 major academic databases—MEDLINE and Embase. In parallel, we systematically reviewed key clinical trial registries, including the EU Clinical Trials Register, the U.S. ClinicalTrials.gov database, and the WHO International Clinical Trials Registry Platform (ICTRP). To ensure maximal comprehensiveness and to identify basket trials not captured through conventional academic databases or clinical trial registries, we employed two complementary web-based search strategies Google Scholar (Academic Grey Literature) and Google Web Search (General Grey Literature).

We anticipated that this multi-source strategy would identify the majority of basket trials relevant to RDs research; however, we restricted our inclusion to studies published in English, which may have excluded trials reported exclusively in other languages such as Chinese or Japanese.

### Systematic research tools

#### Academic databases publications: MEDLINE and Embase

We used the same keywords — basket trial and rare disease — in both MEDLINE and Embase to ensure consistency across searches. Given the anticipated small number of basket clinical trials related to rare diseases, we deliberately chose not to apply exclusion criteria regarding publication year, comparators, interventions, or outcomes. This inclusive approach was intended to maximise sensitivity and avoid the risk of omitting potentially relevant studies in this emerging field. All records retrieved from each database were screened fully. Citations were imported into Zotero, where duplicate records were automatically identified and verified by manual review before screening, and trials reported across multiple publications were consolidated into single study entries. The detailed results are presented in Table 1.

**Table 1:** Selected Study Methodologies from Medline and Embase

Publications academic databases	MEDLINE	Embase
Key words used	Basket Trial and rare disease	Basket Trial and rare disease
Number of items	75	165
Apply Filters	- 74 (English) - 59 (Human) - 24 (Clinical Trial)	- 105 (Clinical Trial)
Excluded items	13: • 7 Redundant Studies • 6 Off-Topic Studies	91: • 8 studies already identified in Medline • 55 redundant publications • 7 off-topic publications • 17 studies not classified as basket trials • 4 basket trials unrelated to rare diseases/cancers
Number of selected studies	11	14

#### Clinical studies database

Additionally, we searched for basket trials in the field of rare diseases across three major international clinical trial registries and databases — the U.S. ClinicalTrials.gov database, the EU Clinical Trials Register, and the WHO International Clinical Trials Registry Platform (ICTRP) — using the same keywords (Basket Trial and rare disease). The results are presented in Table 2.

**Table 2:** Selected Study Methodologies from Four Clinical Trial Databases

Clinical trial registries and databases	The US clinical trial Gov. database	The EU clinical trials register	ICTRP
Key words used	Basket Trial and rare disease	Basket Trial and rare disease	Basket Trial and rare disease
Number of items	13	1	1
Excluded items	8: • 4 already indexed • 4 Off-Topic Studies	0	0
Number of selected studies	5	1	1

#### Web-based search strategies

To broaden the scope of our systematic review and capture studies not indexed in traditional clinical trial databases, we conducted complementary web-based searches using both Google Scholar and Google Web Search. In both cases, we applied the exact phrase “basket trial and rare disease clinical study”, restricting results to the period from 2013 to 2025. For Google Scholar, this approach targeted academic grey literature such as theses, conference abstracts, preprints, or institutional reports not listed in MEDLINE, Embase, or clinical trial registries. For Google Web Search, the same strategy was applied to identify general grey literature, including white papers, regulatory announcements, or press releases. The review of Google Scholar and Google Web Search results was limited to the first 200 hits, as preliminary scoping searches indicated that the relevance of results markedly decreased beyond this point, making a comprehensive review of all results impractical and unlikely to yield additional pertinent information. The results can be found in Table 3.

**Table 3:** Selected Study Methodologies from Google Scholar and Google Web Search

Web-based search	Google Scholar	Google
Key words used	basket trial and rare disease clinical study	basket trial and rare disease clinical study
Number of items	67 200	> 3 million
Apply Filters	Between 2013 and 2025: 17 700 Selection of the first top 200 most relevant items	– Verbatim search (using quotation marks for exact phrase matching): > 3 million Selection of the first top 200 most relevant items
Excluded items	199: • 149 Not a clinical study: Review, Website, Regulatory/Guidance, etc.: • 15 Not a basket trial: • 17 Already indexed • 15 duplicates • 3 not in rare diseases	197: • 128 Not a clinical study: Review, Website, Regulatory/Guidance, etc • 7 not basket trials • 3 not in rare diseases • 22 already indexed • 30 duplicates • 7 off-topic
Number of selected studies	1	3

### Data extraction and synthesis

From each included study, we extracted:

- Trial name and publication reference
- Targeted conditions
- Study design: Trial phase, design features (randomization, control group)
- Recruitment scope (centers, countries)
- Clinical trial number
- Drug used
- Primary endpoints
- Trial status (ongoing/completed) (Date starting and completed)
- Ages Eligible for Study
- Study duration

## Results

### Academic databases Publications: MEDLINE and Embase

In MEDLINE, we identified 24 clinical studies. After removing duplicates and consolidating multiple reports of the same trial, 11 distinct basket clinical trials were retained, the majority targeting rare cancers.

Embase yielded 105 clinical studies. Following the same screening and deduplication process, 14 distinct basket clinical trials relevant to rare diseases were identified.

The results are summarized in Table 1.

### Clinical studies database

In the U.S. ClinicalTrials.gov database, we identified 13 records. Eight were excluded, including four already indexed in other sources and four off-topic studies. The remaining five basket clinical trials were retained.

In the EU Clinical Trials Register, we identified one record, which met the inclusion criteria and was retained.

In the WHO International Clinical Trials Registry Platform (ICTRP), we identified one record, which also met the inclusion criteria and was retained.

The results are summarized in Table 2.

### Web-based search strategies

In Google Scholar, the search returned 17,700 results (from 67,200 total items in the date range 2013–2025). We screened the first 200 entries and excluded 199 items (149 not clinical studies, 15 not basket trials, 17 already indexed, 15 duplicates, and 3 not related to rare diseases). One basket trial relevant to rare diseases was retained.

In the Google Web Search, the query returned more than 3 million results. We screened the first 200 entries and excluded 197 items (128 not clinical studies, 7 not basket trials, 3 not related to rare diseases, 22 already indexed, 30 duplicates, and 7 off-topic). Three basket trials relevant to rare diseases were retained.

The detailed results are presented in Table 3.

In our systematic search, we identified 36 basket trials investigating rare diseases, including rare cancers, across multiple databases and registries. We retrieved most trials from MEDLINE, with 11 trials, and from Embase, with 14 trials. Additional trials were identified in ClinicalTrials.gov (five trials), the EU Clinical Trials Register (one trial), the ICTRP registry (one trial), Google Scholar (one trial), and Google Web Search (three trials).

Table 4 summarizes the results of the systematic review.

**Table 4:** Basket trials and rare disease- results of research by different clinical trial registries and databases

References	Study acronym	Targeted rare diseases	Study Design	Country	Trial ID	Drug	Primary outcome
**MEDLINE**							
[32]	AcSé	Rare cancers incl. sarcomas, ovarian, CNS, thyroid, etc	Multicenter, non-randomized, open-label, single-arm, Phase II	France	NCT03012620	Pembrolizumab	objective response rate
[33]	ROAR	BRAFV600E-mutant low-grade and high-grade glioma [rare cancer)	Multicentre, open-label, single-arm, phase 2	13 countries [Austria, Belgium, Canada, France, Germany, Italy, Japan, Netherlands, Norway, South Korea, Spain, Sweden, USA)	NCT02034110	Dabrafenib plus trametinib	objective response rate
[34]	DART	Nonpancreatic Neuroendocrine Tumors	Multicentre, open-label, Parallel -Assignment, phase 2	USA	NCT02834013	Ipilimumab plus nivolumab	overall response rate
[35]	NC	Advanced epithelioid sarcoma with loss of INI1/SMARCB1	Multicentre, open-label, single Assignment, phase 2	Australia, Belgium, Canada, France, Germany, Italy, Taiwan, USA, UK	NCT02601950	Tazemetostat	overall response rate
[36]	NC	Rare Tumors That Cannot Be Removed by Surgery or Are Metastatic	Monocentre, open-label, single-arm, phase 2	USA	NCT02721732	Pembrolizumab	Non-progression Rate (NPR)
[37]	JUPITER	locally advanced or metastatic, solid cancers harboring HER2 amplification	Multicentre, open-label, single-arm, phase 2	Japan	jRCT2031180150	Trastuzumab and pertuzumab	objective response rate
[38]	BIVV009-01	Complement-mediated Disorders, cold agglutinin disease	Double-blind, randomized, placebo-controlled basket trial, Phase 1b	Austria	NCT02502903	BIVV009: monoclonal antibody directed against the C1s subunit of human complement component C1	safety and tolerability of BIVV009
[39]	PARAGON	Women with Estrogen or Progesterone Receptor-Positive Platinum-Resistant or -Refractory Recurrent Ovarian Cancer	Multicentre, open-label, single-arm, phase 2	United Kingdom, New Zealand, Belgium	ACTRN12610000796088	Anastrozole	clinical benefit (complete/partial response + stable disease) rate (CBR)
[40]	AcSé2	Rare Advanced cancers trichoblastic carcinomas	Multicentre, open-label, single-arm, phase 2	France	NCT03012581	Nivolumab (anti-PD1 immunotherapy)	objective response rate
[41]	NC	Advanced rare solid tumors	Monocentre, open-label, single-arm, phase 2	USA	NCT02938793	Durvalumab + tremelimumab	antitumor activity, Safety
[42]	VE-BASKET	BRAF V600 Mutation-positive Cancers	Open-label, multicohort, Phase II,	US, China, France, Germany, Spain, UK	NCT01524978	Vemurafenib, cetuximab	objective response rate
**EMBASE**							
[43]	DRIMID	Behçet’s disease, idiopathic inflammatory myopathies and IgG4-related disease	Multicentre, phase II, single-arm uncontrolled, non-randomized trial, Open label	Netherlands	NCT06285539	Filgotinib	EQ-5D-5L change, Disease activity for each pathology
[44]	NC	Solid Tumor Cancers; Lung Cancer; Bladder Cancer; Urinary Tract Cancers	Multicentre, phase II, Parallel Assignment uncontrolled, non-randomized trial, Open label	USA	NCT02675829	Ado-trastuzumab emtansine	best overall response (ORR)
[45]	CABATEN	Neuroendocrine Tumor; Anaplastic Thyroid Cancer: Adenocarcinoma; Pheochromocytoma; Paraganglioma	Multicentre, phase II, single Assignment uncontrolled, non-randomized trial, Open label	Spain	NCT04400474	Cabozantinib; Atezolizumab	objective response rate
[46]	CIELO	NMDAR Autoimmune Encephalitis; LGI1 Autoimmune Encephalitis	Multicentre, Phase 3, randomized, double-blind, Placebo-controlled, basket study	USA, Argentina, Austria, Brazil, China, Czechia, Denmark, France, Ghana, italy, Japan, Korea, Netherlands, Poland, Taiwan	NCT05503264	Satralizumab	Proportion of Participants in NMDAR AIE Cohort With Modified Rankin Scale (mRS) Score Improvement ≥ 1
[47]	ABNL-MARRO	myelodysplastic; myeloproliferative neoplasms	Mono center, Phase 1/2, Single Group Assignment, open label	USA	NCT04061421	Itacitinib; ASTX727	Recommended phase 2 dose (Phase I), Dose-limiting toxicity (Phase I), Overall response (Phase II)
[48]	PEVOsq	Squamous Cell Lung Cancer; Vulvar Cancer; Penile Cancer; Cervix Cancer; Head and Neck Squamous Cell Carcinoma; Anal Cancer	Open-label, non-randomized, multi-center, Single Group Assignment, phase II trial,	France	NCT04357873	Pembrolizumab; vorinostat	Objective Response Rate (ORR)
[49]	Zuma-25	Relapsed/Refractory Waldenstrom Macroglobulinemia; Relapsed/Refractory Richter Transformation; Relapsed/Refractory Burkitt Lymphoma; Relapsed/Refractory Hairy Cell Leukemia	Phase 2, non-randomized, Open-Label, Multicenter, Parallel Assignment	Austria, France, Germany, Italy, Netherlands, Spain, Switzerland, United States	NCT05537766	Cyclophosphamide, Fludarabine, Brexucabtagene Autoleucel	Substudy A: Combined Rate of Complete Response (CR) and Very Good Partial Response (VGPR) Substudy B, C, and D: Objective Response Rate (ORR)
[35]	NC	Malignant Rhabdoid Tumors (MRT); Rhabdoid Tumors of the Kidney (RTK); Atypical Teratoid Rhabdoid Tumors (ATRT); Selected Tumors With Rhabdoid Features; Synovial Sarcoma; INI1-negative Tumors; Malignant Rhabdoid Tumor of Ovary; Renal Medullary; Any Solid Tumor with an EZH2 GOF Mutatio Carcinoma;Poorly Differentiated Chordoma (or Other Chordoma with Sponsor Approval) Epithelioid Sarcoma	Phase II, non-randomized multicenter, open-label, single arm, phase 2	Australia, Belgium, Canada, France, Germany, Italy, Taiwan, United Kingdom, United States	NCT02601950	Tazemetostat	Cohorts 1, 3, 4, 5, 6 and 7: Objective Response Rate (ORR) Cohort 2: Progression-Free Survival (PFS) Cohort 8:Safety
[50]	NC	Glioma; Cholangiocarcinoma; Solid Tumor; IDH Mutation	Phase 2, non-randomized, Open-Label, Mono center, Parallel Assignment	Canada	NCT03991832	Olaparib, Durvalumab	Overall response rate
[51]	SUMMIT	Solid Tumors Harboring Somatic HER2 or EGFR Exon 18 Mutations	Phase 2, non-randomized, Open-Label, Multicenter, Parallel Assignment	Australia, Belgium, Canada, Denmark, France, Ireland, Israel, Italy, Korea, Republic of, Serbia, Spain, United Kingdom, United States	NCT01953926	Neratinib, Fulvestrant, Trastuzumab, Paclitaxe	Objective Response Rate (ORR)
[52]	ROCK	advanced rare cancers with dMMR/MSI-H	Phase II, non-randomized multicenter, open-label, single arm,	Japan	JMA-IIA00344	Nivolumab	Objective response rate
[53]	STARTRK-2	Breast Cancer; Cholangiocarcinoma; Colorectal Cancer; Head and Neck Neoplasms; Lymphoma; Large-Cell; Anaplastic; Melanoma; Neuroendocrine Tumors; Non-Small Cell Lung Cancer; Ovarian Cancer; Pancreatic Cancer; Papillary Thyroid Cancer; Primary Brain Tumors; Renal Cell Carcinoma; Sarcomas; Salivary Gland Cancers; Adult Solid Tumor	Phase II, non-randomized multicenter, open-label, Parallel Assignment	Australia, Belgium, China, France, Germany, Hong Kong, Italy, Japan, Korea, Republic of, Netherlands, Poland, Singapore, Spain, Taiwan, United Kingdom, United States	NCT02568267	Entrectinib	Objective response rate
[54]	My Pathway	Neoplasms; Solid Tumors; Biliary Cancer; Salivary Cancer; Bladder Cancer	Phase II, non-randomized multicenter, open-label, Parallel Assignment	USA	NCT02091141	Trastuzumab, Pertuzumab, Vemurafenib, Cobimetinib, Vismodegib, Alectinib, Atezolizumab	Objective Response Rate (ORR)
[55]	NAVIGATE	Solid Tumors Harboring NTRK Fusion	Phase II, non-randomized multicenter, open-label, Parallel Assignment	Argentina; Australia, Belgium, Brazil, Canada, China, Colombia, Czechia, Denmark, France, Germany, India, Ireland, Italy, Japan, Korea, Republic of, Portugal, Russian Federation, Singapore, Slovakia, Spain, Sweden, Taiwan, Turkey, United States	NCT02576431	BAY2757556 (Larotrectinib, Vitrakvi)	Best Overall Response or partial response (PR)
**Clinical Trial.Gov database**							
[56]	ANTARES	Advanced or metastatic rare tumors	Phase II basket trial, single-arm trial, Open label	Brazil	NCT06638931	Nivolumab (monoclonal anti-PD1 antibody)	disease control rate
[57]	DETERMINE	Cancers With Actionable Genomic Alterations, Including Common Cancers with Rare Actionable Alterations	Umbrella-Basket Platform Trial, Phase II/III Non-Randomized, Single Group Assignment	UK	NCT05722886	Alectinib, Atezolizumab, Entrectinib, Vemurafenib	Objective Response Rate (ORR) and Durable Clinical Benefit
[58]	CRAFT	BRAF V600E/K, (ii) ERBB2 amplification and/or overexpression or activating ERBB2 mutation	7-arm basket phase II clinical trial, Non-Randomized, Parallel Assignment	Germany	NCT04551521	Cobimetinib, Atezolizumab,Trastuzumab,Alectinib, Ipatasertib,Inavolisib	Disease control rate
[59]	NADAPT	Progressive Supranuclear Palsy; Multiple System Atrophy; Corticobasal Syndrome	Randomized, double blinded, phase II trial, follows a basket trial design	Norway	NCT06162013	Nicotinamide Riboside	PSP Cohort: PSP Rating Scale; MSA Cohort: Unified MSA Rating score; CBS Cohort: Between group difference in PSP Rating Scale (PSPRS) total score
[60]	LHON-Plus	Mitochondrial Encephalopathy, Lactic Acidosis, Stroke-like Episodes (MELAS) or Leber's Hereditary Optic Neuropathy-Plus (LHON-Plus)	Non-randomized dose-escalation, open-label basket exploratory phase 1,	USA	NCT06792500	Glycerol Tributyrate	safety and efficacy of Glycerol Tributyrate
**The EU clinical trial register**							
[61]	RM-493	Raregenetic disorders of obesity and hyperphagia	Phase 2 open-label, uncontrolled, proof-of-concept, basket study	Canada, France, Germany, Greece, Israel, Netherlands, Spain, UK, USA	NCT03013543	Setmelanotide	The proportion of patients in each subgroup who achieve at least 5% body weight reduction
**ICTRP**							
[62]	ORBCEL-C	Primary sclerosing cholangitis, rheumatoid arthritis, lupus nephritis and Crohn’s disease	A single-arm, multi-centre, Non-randomized; Interventional; Open label, Phase II basket trial	UK	NCT02997878	Cell therapy made of Mesenchymal Stromal Cells (MSC)	Dose finding and incidence of treatment emergent adverse events
**Google scholar**							
[63]	CREATE	Advanced Tumors Induced by Causal Alterations of ALK and/or MET	Multicenter, Phase 2, Non-Randomized, Parallel Assignment, Open-label Basket Study	Belgium, France, Germany, Italy, Netherlands, Norway, Poland, Slovakia, Slovenia, UK	NCT01524926	Crizotinib	Antitumor activity
**Google web search**							
[64]	INTUITT-NF2	Neurofibromatosis Type 2; Vestibular Schwannoma; Non-vestibular Schwannoma; Meningioma; Ependymoma	Randomized, Open Label, parallel Assignment, multi-arm phase II platform-basket screening study designed to test multiple experimental therapies simultaneously in patients with NF2-related schwannomatosis	UK	NCT04374305	Brigatinib, Neratinib	Radiographic response rate
[65]	REAL 9	Turner syndrome, Noonan syndrome, or idiopathic short stature	Controlled, Randomised, Open label, Parallel group, basket trial, Phase III	Malaysia, Switzerland Brazil, Canada China, India Israel, Japan Korea, Republic of Mexico Russian Federation Saudi Arabia, Serbia South Africa, Thailand United Kingdom, United States European Union	NCT05723835	Somapacitan	Height velocity reported, safety
[66]	FORTITUDE-301	Solid Tumors with Fibroblast Growth Factor Receptor 2b (FGFR2b]	Multicenter, Phase 1b/2, Non-Randomized, Open-label Basket Study	USA	NCT05325866	Bemarituzumab	Objective Response (OR) Rate, Safety

A more detailed table summarizing these different studies can be found in Supplementary Table 1.

### Trial focus

Of the 36 basket trials included in the review, the majority (27 trials, 75%) focused on rare cancers, while only nine addressed non-oncological rare diseases. These nine non-oncology trials collectively encompassed 25 distinct RDs, with no pathological overlap observed across studies. Notably, 11 of the included basket trials explored therapeutic interventions for three or more RDs, while the remaining 25 trials focused on one or two conditions, reflecting a varied scope in the application of basket trial methodology across the rare disease spectrum. Two examples illustrate the breadth of this approach. The NADAPT trial is a multi-arm study designed to evaluate targeted therapies across a spectrum of ultra-rare neurodevelopmental disorders, grouping patients by underlying genetic mechanisms rather than by clinical phenotype [59]. Similarly, the INTUITT-NF2 study assessed the efficacy of brigatinib in patients with neurofibromatosis type 2 (NF2)–related tumors, including vestibular schwannomas, non-vestibular schwannomas, meningiomas, and ependymomas, exemplifying how a single therapeutic strategy can be applied across multiple tumor types within the same RD spectrum [64]. These studies demonstrate how basket designs can accommodate considerable heterogeneity while remaining molecularly or mechanistically coherent.

### Trial status and phases

At the time of our review, 15 of the 36 identified basket trials had been completed, while 21 were still ongoing. Most of these trials were classified as Phase II (29 studies, 81%), underscoring the exploratory nature of basket designs in RDs research. Early-phase trials were less frequent, with two studies (6%) conducted as Phase I and two others (6%) structured as combined Phase I/II investigations. Later-phase trials were comparatively rare, with only one trial categorized as Phase II/III and two studies (6%) reaching Phase III.

### Study design and methodology

The predominant trial design among the 36 included studies was non-randomized and open-label, accounting for 31 trials (86%). Only five studies (14%) incorporated randomization, of which three were double-blinded and two were open-labelled. Regarding allocation methods, parallel assignment was employed in 19 trials (53%), while 16 trials (44%) followed a single-arm design. Most studies were conducted across multiple centers, with 32 trials (89%) identified as multicentric and only four limited to a single center. The average number of participating centers per trial was 56, with the highest number 1,013 centers, reported in the Dual Anti-CTLA-4 and Anti-PD-1 Blockade in Rare Tumors (DART) study targeting neuroendocrine tumors. Among the nine basket trials addressing non-oncological RDs, the average number of centers was notably lower, at 24, with the study of satralizumab in patients with NMDAR-IgG-antibody-positive or LGI1-IgG-antibody-positive autoimmune encephalitis (CIELO study) involving the largest number of sites at 81.

### Treatment strategies

Of the 36 basket trials included in the review, 23 studies (64%) evaluated monotherapy regimens, whereas the remaining 13 trials (36%) investigated combination therapies.

### Trial registration and geographical distribution

Among the 36 basket trials analyzed, the vast majority (32 trials) were registered on ClinicalTrials.gov, the U.S. clinical trial registry, and were assigned NCT identification numbers. The remaining four trials were recorded in other international databases: two in Japanese registries (JRCT and JMA) and two in the EudraCT (European Union) and ACTRN (Australia/New Zealand) registries. Regarding geographical scope, 11 trials were multinational—primarily spanning the United States and Europe—while the remaining 25 were conducted within a single-country framework.

### Endpoint selection

The analysis of primary endpoints revealed marked differences between oncological and non-oncological RDs basket trials. Among the nine non-oncology trials, there was substantial heterogeneity in the choice of primary endpoints—primarily clinical criteria—with no single endpoint consistently used across all studies. In contrast, basket trials targeting rare cancers demonstrated greater uniformity, with the Objective Response Rate (ORR) selected as the primary endpoint in 22 out of 27 studies (81%).

### Study Duration

The average duration of the basket trials included in the review was 6.55 years. Trial durations ranged from a minimum of 1 year, as observed in the Leber's Hereditary Optic Neuropathy-Plus (LHON-Plus) study targeting neuropathies [61], to a maximum of 11 years for study NCT02938793, which focused on advanced rare solid tumors [41]. The earliest recorded basket trial began in 2012 and targeted BRAF V600 mutation-positive cancers (VE-BASKET) [43]. Among the nine non-oncological RDs trials, the earliest identified study commenced in 2015 (BIVV009-01) [38], marking the starting point for basket trial applications in this subset of RDs.

### Patient enrolment

The mean number of patients enrolled per basket trial was 203, ranging from 20 to 825, with a median of 154. The mean was strongly influenced by large outliers, particularly the DETERMINE trial (Determining Extended Therapeutic Indications for Existing Drugs in Rare Molecularly Defined Indications Using a National Evaluation Platform), a large Phase 3 study focusing on cancers with rare actionable alterations [57].

Among the 36 basket trials analyzed, 28 (77%) were restricted to adults, while the remaining 8 (22%) included pediatric populations. The minimum eligible age varied across these pediatric-inclusive trials. Two studies—the EORTC Phase II 90101 CREATE trial and the DETERMINE study—enrolled patients aged one year or older [57, 63]. The Setmelanotide (RM-493) study included participants aged six years and above, and the REAL 9 study accepted individuals between 10 and 18 years [61]. Additionally, two trials—the INTUITT-NF2 study (Innovative Trial for Understanding the Impact of Targeted Therapies in NF2-Related Schwannomatosis) and the CIELO study—set the minimum age at 12 years [46, 64], while the AcSé (INCa “secured access”) trial and the VE-BASKET study permitted enrollment from ages 15 and 16, respectively [32, 43].

## Discussion

This systematic review provides a comprehensive evaluation of the use of basket trials in RDs while highlighting three principal findings: their main use in rare oncological diseases, their limited use in non-oncological RDs, and the critical barriers hindering broader basket trial implementation. While molecularly-driven trial designs have revolutionized rare cancer research, their translation beyond oncology is constrained by heterogeneous disease mechanisms, underdeveloped biomarkers, and the absence of standardized therapeutic frameworks. Addressing these challenges is crucial to harnessing the full potential of basket trials in diverse RDs contexts.

### Rare oncology’s leadership in basket trial adoption

The overwhelming predominance of basket trials in rare oncology (75%) suggests that this trial design is particularly well-suited to cancer research. In oncology, the classification of tumors based on molecular alterations rather than tissue of origin has facilitated the implementation of basket trials, enabling more efficient drug development and contributing to greater regulatory acceptance [67]. This approach aligns with initiatives such as the U.S. RACE for Children Act, which empowers the FDA to require pediatric evaluation of cancer drugs when the molecular targets are relevant to childhood cancers, regardless of the drug's initial indication in adults [68].

By contrast, basket trials remain exceedingly rare in non-oncological RDs, largely due to several persistent and interrelated barriers. These include a limited number of validated molecular targets, a lack of reliable biomarkers, an absence of well-established and disease-relevant clinical endpoints—particularly when compared to oncology—as well as a general scarcity of preclinical data and natural history studies. Regulatory uncertainties surrounding trial design and endpoint selection further complicate implementation [69]. The distribution of nine identified non-oncology basket trials across 25 distinct diseases—with no condition overlap—illustrates both the remarkable heterogeneity in this space and the inherent difficulty in identifying shared pathogenic mechanisms that could justify a basket trial approach [69].

The predominance of basket trials in oncology also reflects differences in funding and commercial incentives. Oncology benefits from substantial investment and strong market potential, whereas non-oncological RDs attract less commercial interest due to smaller patient populations and limited return on investment. This disparity is mirrored in the orphan drug landscape, where most approvals to date concern oncology therapies.

### Trial phases and progression

The predominance of Phase II trials (81%) highlights the exploratory nature of basket trials in RDs, aiming to assess preliminary efficacy signals across multiple conditions. Early-phase investigations (Phase I, 6%) and confirmatory Phase III trials (6%) remain markedly underrepresented, reflecting both the nascent application of this trial design in RDs and the challenges associated with its progression to later stages. These findings mirror trends observed in oncology: a recent systematic review reported that 73% (107/146) of oncology basket trials were Phase II, 23% were conducted as dose-expansion cohorts within Phase Ib or I/II trials, and only 3% reached a Phase II/III confirmatory stage [70]. Other reviews corroborate these trends; Park et al. noted that most oncology basket trials remained in early development phases (I or II) [71].

The limited number of Phase III basket trials in RDs (6% in our analysis) suggests that, despite their conceptual appeal, these designs have not yet been widely adopted for confirmatory research. Several factors may contribute to this trend: persistent regulatory uncertainties regarding evidentiary standards for basket trial approvals, the considerable logistical and financial burden of conducting multicenter late-phase trials in RDs, and the methodological difficulty of powering confirmatory studies across heterogeneous conditions. These barriers are compounded by the complexity of RD phenotypes, which often lack shared, validated clinical endpoints and require disease-specific natural history data to support trial design and interpretation.

By contrast, late-phase basket trials have been more feasible in oncology. This difference can be attributed to several structural and scientific advantages: simpler and well-validated endpoints (e.g. objective response rate), greater availability of supportive preclinical and natural history data, and a research focus on shared molecular alterations (e.g. specific oncogenic mutations in cancer cells), which contrasts with the often diverse and multi-system clinical presentations observed in non-oncological RDs. Furthermore, oncology benefits from mature infrastructure—including patient registries, referral networks, and specialized centers— that greatly facilitates patient recruitment. Biostatistical modeling techniques tailored for oncology basket trials have also gained regulatory familiarity, further enabling their use in confirmatory settings.

In non-oncological RDs, however, the relative lack of validated biomarkers and common endpoints continues to limit the feasibility of confirmatory basket trials. As a result, basket trial designs currently remain better suited for early-phase exploratory research in this field [72]. Nonetheless, the growing number of ongoing RD basket trials (21 active studies) reflects increasing interest and momentum. Strengthening natural history data collection, endpoint validation, and regulatory engagement will be essential to support the transition of these trials toward later-phase development and eventual clinical and regulatory adoption.

### Study design consideration

Non-randomized, open-label designs dominate basket trials in RDs, accounting for 86% of studies. While these designs may be pragmatic in the context of small, dispersed patient populations, they raise important concerns regarding bias, reproducibility, and external validity. This mirrors trends observed in oncology, where single-arm studies remain prevalent in early development phases [70, 71]. Haslam et al. further emphasized the field’s dependence on single-arm methodologies, underscoring their limitations in providing robust comparative evidence [73]. The absence of standardized control groups—coupled with heterogeneity in disease progression, clinical endpoints, and treatment response across rare disease subgroups—further complicates interim analyses and limits the strength of efficacy assessments.

Although open-label, non-randomized designs facilitate faster enrollment in rare and ultra-rare disease contexts, they also introduce significant methodological and ethical challenges. These include increased risk of selection bias, potential overestimation of treatment effects, and lack of a meaningful comparator, which collectively limit the generalizability and regulatory interpretability of results. From an ethical standpoint, the absence of control arms may be seen as acceptable—or even necessary— particularly in ultra-rare diseases, where patient numbers are extremely limited and withholding a potentially beneficial treatment is not justifiable. Nevertheless, uncertainty remains within both the expert medical community and regulatory authorities regarding the extent to which outcomes from such trials can reasonably be accepted as demonstrating safety and efficacy. This dilemma is particularly acute in pediatric populations, where most genetic RDs manifest, and where the tension between timely access to innovative therapies and ensuring robust evidence of safety and effectiveness is especially pronounced. In this context, non-randomized basket trials must be carefully designed with rigorous methodological safeguards, transparent reporting, and, when appropriate, post-marketing evidence requirements to balance urgent patient needs with the protection of vulnerable populations [74].

Despite these constraints, 89% of the reviewed basket trials were multicentric, averaging 56 study sites, reflecting the extensive collaborative infrastructure required to overcome recruitment challenges. Large-scale initiatives such as the DART trial, which enrolled 818 patients across over 1,000 sites, exemplify the considerable logistical demands involved in conducting these studies at scale [34].

However, randomization and blinding were implemented in only 14% of the trials reviewed—underscoring the need for enhanced methodological rigor. Future basket trial designs should prioritize innovative and flexible approaches that preserve scientific validity while remaining feasible in RD settings. These may include adaptive trial frameworks, Bayesian statistical models, synthetic or historical control arms, and seamless phase I/II or II/III transitions. Such strategies can improve the interpretability of outcomes while respecting the ethical and logistical constraints inherent to RD research.

### Methodological challenges in basket trial implementation

Conducting basket trials in RDs entails several methodological obstacles, particularly when defining subgroups and achieving adequate statistical power—challenges that become even more critical in late-phase trials, where regulatory expectations are more stringent. These issues are particularly pronounced in the ultra-rare disease context, where foundational data are often missing: preclinical evidence is limited, biomarkers are rarely validated, phenotypic presentations vary widely, and natural history studies are frequently incomplete or unavailable [75]. Together, these gaps hinder the establishment of clear inclusion criteria and complicate the selection of relevant and measurable endpoints.

The heterogeneity of clinical manifestations—sometimes even among individuals with the same genetic mutation—introduces noise that can obscure treatment effects and reduce the interpretability of outcomes. Additionally, in many rare conditions, there is no consensus on validated endpoints, which further complicates cross-subgroup comparisons and weakens the strength of efficacy assessments [76]. These limitations are particularly problematic in confirmatory trials, where regulatory agencies require a higher level of evidence and methodological rigor.

Furthermore, because basket trials typically involve testing a single therapeutic across multiple disease entities, ensuring adequate statistical power for each subgroup is often difficult. The need for multiplicity adjustments and increased sample sizes strains the feasibility of such trials, especially when patient populations are extremely small. In this context, traditional statistical approaches may fall short, reinforcing the need for more flexible methods—such as hierarchical or Bayesian modeling—that can borrow strength across related groups while maintaining interpretability.

To address these challenges, trial designs must incorporate thoughtful, pre-specified subgroup analyses and leverage innovative statistical and operational approaches. Adaptive designs, seamless phase transitions, and the use of external or synthetic control arms are examples of strategies that may help optimize data generation without undermining feasibility. Ultimately, advancing the use of basket trials in RDs will depend on continued methodological innovation, combined with early and proactive engagement with regulators to ensure the acceptability of trial designs and endpoints.

### Study timelines and implementation challenges

The average duration of the basket trials included in our review was 6.55 years, with timelines ranging from as short as 1 year—for example, in the LHON-Plus study targeting hereditary optic neuropathies [61]—to as long as 11 years in the case of the INTUITT-NF2 trial [64]. This latter study, evaluating bevacizumab in patients with neurofibromatosis type 2–related schwannomatosis (NF2-SWN), illustrates the prolonged timelines often required for complex, multicenter trials in rare disease populations, where recruitment is slow and follow-up periods are typically long.

Although basket trials have been in development for over a decade, their expansion has been slow and uneven. Several factors appear to have contributed to this limited uptake. These include a relative lack of engagement from pharmaceutical sponsors—often deterred by the fragmented and small patient populations—and a certain degree of conservatism among clinicians and academic researchers, who may hesitate to adopt non-traditional trial designs. The earliest trial identified in our dataset began in 2012, targeting cancers with BRAF V600 mutations [43]. In contrast, the first non-oncological basket trial—the BIVV009-01 study—was not launched until 2015 [38], underscoring a lag in the adoption of basket methodologies outside the oncology field.

Taken together, these observations highlight both the promise and the complexity of basket trials in RDs. While this design offers a flexible framework to accelerate therapeutic development, especially when targeting shared molecular mechanisms, it also demands careful long-term planning and robust infrastructure to address the operational and scientific challenges inherent to these trials.

### Treatment strategies and endpoint selection

Our analysis reveals distinct trends in therapeutic strategies and outcome measures across basket trials, highlighting key methodological considerations for their application in RDs. The predominance of monotherapy investigations (64%) reflects the core principles of precision medicine, wherein targeted single-agent therapies are matched to specific molecular alterations. Conversely, the limited exploration of combination therapies (36%) suggests the inherent complexities of multi-target approaches, particularly in non-oncological RDs, where multifactorial pathophysiology complicates therapeutic development and trial design [69].

A critical distinction between oncological and non-oncological basket trials lies in endpoint selection. Oncology trials exhibit notable consistency, with Objective Response Rate (ORR) serving as the primary endpoint in 81% of studies—a trend well-documented in prior systematic reviews [70, 71, 73]. This uniformity can be attributed to the well-established biomarker-response correlation in oncology [77, 78], the structured evolution of therapeutic development pathways for malignant conditions, and the regulatory precedence supporting ORR as a surrogate for clinical benefit [79]. In contrast, non-oncological rare disease trials display significant heterogeneity in outcome measures, reflecting ongoing challenges in defining validated efficacy endpoints for these diverse conditions [80].

This variability in endpoint selection poses substantial obstacles for comparative effectiveness research, regulatory decision-making, and the translation of trial results into clinical practice. To address these challenges, we advocate for a structured yet adaptable framework to improve endpoint harmonization across rare disease basket trials. Building on previous recommendations, we propose a multi-pronged strategy:

- **Development of disease-specific core outcome sets:** This should be achieved through Delphi consensus processes to ensure alignment with clinical priorities and regulatory expectations [81]. Patient advocacy organizations, in close collaboration with clinical experts and regulators, are well-positioned to lead this effort, given their ability to represent patient priorities and mobilize consensus across diverse stakeholders.
- **Integration of composite endpoints**:: Combining functional outcomes, surrogate biomarkers, and patient-reported outcome measures (PROMs) can better capture the multidimensional nature of treatment benefit [82]. The use of surrogate endpoints is particularly important in this context, especially when long-term outcomes are not readily measurable. PROMs can be drawn from validated repositories such as PROQOLID (Patient-Reported Outcome and Quality of Life Instruments Database) [83], which provides access to a wide range of standardized and validated instruments, or developed through initiatives such as COMET (Core Outcome Measures in Effectiveness Trials), which offers structured methodologies for defining core outcome sets and ensuring patient-centered relevance [84].
- **Incorporation of novel digital endpoints:** Supported by wearable technologies and continuous monitoring tools. Notable examples include the EMA-validated SV95C wearable sensor, which quantifies changes in ambulatory function by measuring stride velocity in natural walking conditions. This objective and continuous physiological measurement offers enhanced sensitivity to detect clinically meaningful changes over shorter timeframes, providing particular value in rare conditions where conventional outcome measures are often impractical [85].
- **Leveraging real-world evidence (RWE):** To complement traditional clinical endpoints and support regulatory decision-making. Existing regulatory platforms and academic repositories, such as Data Analysis and Real World Interrogation Network (DARWIN-EU) [86], provide valuable infrastructures for systematic RWE collection and analysis. Collaborative efforts among academic consortia, regulatory bodies, and patient registries are essential to ensure data quality, standardization, and applicability.

Implementing these methodological innovations will not only improve the reliability and comparability of basket trial outcomes, but also enhance their clinical and regulatory relevance. By striking a balance between necessary standardization and the flexibility that defines the basket trial design, these strategies may enable broader application beyond oncology and support more efficient therapeutic development in underserved rare disease populations. While these recommendations align with the broader scope of initiatives such as REALISE-D, they also extend beyond it, highlighting areas where multi-stakeholder collaboration is needed to accelerate innovation across the rare disease research landscape

#### Patient recruitment and feasibility in rare disease basket trials

Our analysis reveals considerable variability in patient enrollment across rare disease basket trials, with a mean sample size of 232 participants (range: 20–825). This broad distribution reflects the persistent challenges of recruiting sufficient patients in rare disease research, where populations are not only small but also geographically dispersed and diagnosed through heterogeneous clinical pathways. Although basket trials offer a potential advantage by pooling multiple disease cohorts under a shared therapeutic hypothesis, 27% of the trials analyzed enrolled fewer than 100 participants—raising concerns about statistical power, the reliability of findings, and their generalizability to broader clinical practice [87].

These recruitment challenges are particularly pronounced in non-oncological RDs, where several structural and clinical limitations converge. Many of the conditions studied are ultra-rare, with only a few patients per country or region, making it difficult to identify and enroll adequate numbers within a single jurisdiction. In addition, the lack of centralized care networks and comprehensive patient registries hampers early identification and coordinated referral to clinical sites [88]. The trial infrastructure in non-oncology fields also tends to be less mature than in oncology, with fewer investigator networks, limited funding mechanisms, and reduced institutional experience with complex multicenter protocols. These factors contribute to frequent delays and under-enrollment, thereby limiting both the internal validity and external applicability of the trial findings. Diagnostic delays and heterogeneity in disease progression further exacerbate this issue, as patients may miss the therapeutic window required for inclusion.

Another critical gap revealed by our analysis is the underrepresentation of pediatric populations. Although a substantial proportion of RDs manifest during infancy or early childhood, most basket trials (28 out of 36, or 77%) restricted participation to adults. Only eight studies (22%) explicitly allowed the inclusion of patients under 18 years of age, with minimum age thresholds ranging from 1 to 15 years. This limited representation of children and adolescents is especially concerning given that a substantial proportion of RDs manifest during early childhood. Expanding eligibility to younger age groups is critical for the development of effective pediatric therapies. However, recruiting pediatric patients introduces additional layers of complexity, including stringent ethical requirements, age-specific consent and assent processes, and logistical considerations such as the need for child-friendly facilities and specialized clinical expertise [89].

To overcome the recruitment barriers, we propose a multi-pronged approach that combines evidence-based strategies designed to improve patient recruitment and trial feasibility across rare disease basket trials. These strategies are organized into four key areas:

##### Global collaborative networks

Establishing multinational investigator consortia with standardized protocols is crucial for improving recruitment efficiency across different regions. Furthermore, implementing centralized patient matching systems that span multiple countries will facilitate the identification of eligible patients across borders, broadening trial participation and increasing enrollment [90].

Initiatives such as the EU Cross-Border Clinical Trials Initiative (EU-X-CT), launched under the auspices of the European Forum for Good Clinical Practice (EFGCP) and the European Federation of Pharmaceutical Industries and Associations (EFPIA)) [91], exemplify this collaborative approach. By bringing together patient organizations, academic centers, clinical networks, industry partners, and regulatory authorities, EU-X-CT provides a scalable, interoperable platform tailored to the specific needs of complex, multinational trials, including those based on a basket design. The initiative promotes data harmonization, streamlines regulatory engagement, and enhances site readiness, thereby directly addressing key logistical and methodological barriers to recruitment in rare disease studies. Its structure is particularly well-aligned with the operational demands of basket trials, which often require the parallel inclusion of small patient subgroups across geographically dispersed sites.

In parallel, the European Reference Networks (ERNs)—virtual consortia of expert healthcare providers specializing in rare and complex diseases—offer a powerful clinical and infrastructural backbone for basket trials [92]. ERNs facilitate the exchange of expertise, coordinate care across borders, and support harmonized data collection in real-world clinical settings through the European Rare disease research Coordination and support Action (ERICA) consortium which includes 24 ERNs [93]. Their disease-specific focus and long-standing collaboration mechanisms represent an underutilized asset in clinical trial design and implementation. By actively involving ERNs in trial planning and patient recruitment strategies, sponsors and investigators can benefit from access to curated patient cohorts, centralized diagnostic expertise, and streamlined communication with local clinical teams.

Equally important is the integration of natural history data into trial planning and patient stratification. While quantitative data on disease progression remain a cornerstone of eligibility and endpoint definition, qualitative natural history insights—often derived from longitudinal clinical observation, expert consensus, and patient-reported experiences—play a vital role in understanding disease trajectories, phenotypic variability, and potential sources of heterogeneity. The collection and sharing of these qualitative data across expert networks, such as ERNs and international registries, enhances the capacity to define meaningful clinical endpoints, optimize subgroup allocation, and tailor therapeutic hypotheses to real-world disease manifestations.

Taken together, these collaborative strategies underscore the importance of embedding rare disease basket trials within global, multidisciplinary ecosystems that prioritize data sharing, regulatory alignment, and patient-centered design. Such infrastructures not only expand the geographical and clinical reach of trials but also ensure that study methodologies are rooted in the lived realities and scientific complexities of rare disease populations.

#### Patient-centric recruitment infrastructure

Efficient patient recruitment remains a major challenge in rare disease basket trials. In this context, the strategic use of established rare disease registries—such as RD-Connect, European Organisation for Rare Diseases EURORDIS [94], and the newly launched virtual platform under the European Rare Diseases Research Alliance ERDERA [27]—offers considerable potential for improving patient identification and pre-screening. These centralized platforms enable access to standardized, real-time patient data across multiple countries, facilitating faster and more coordinated recruitment processes.

Equally important is the active involvement of patient advocacy organizations, which play a key role in fostering trust, disseminating information, and encouraging trial participation through community engagement. Their collaboration not only increases trial visibility but also ensures that recruitment strategies are aligned with patient needs and preferences.

In parallel, the development of trial-ready cohorts through longitudinal natural history studies can further streamline enrollment. By pre-identifying individuals who meet key inclusion criteria, these cohorts reduce the administrative and logistical burden of trial initiation, shorten recruitment timelines, and help ensure that trials can progress more efficiently. Together, these approaches form the foundation of a patient-centric recruitment infrastructure that is both pragmatic and responsive to the unique challenges of rare disease research.

#### Innovative data integration

The integration of real-world evidence (RWE) derived from electronic health records (EHRs), wearable digital biomarkers, and patient-reported outcome (PRO) platforms presents a transformative opportunity to enhance patient selection, real-time monitoring, and outcome assessment in rare disease basket trials [95]. These decentralized data sources enable a more dynamic, continuous, and patient-centered approach to trial design, offering timely insights into treatment effectiveness, safety, and variability across diverse patient subgroups.

Among the most promising methodological innovations is the use of synthetic control arms built from well-curated historical trial datasets or longitudinal natural history cohorts. In the context of RDs—where small sample sizes, fragmented populations, and ethical constraints frequently preclude traditional randomized control groups—synthetic controls provide a scientifically robust alternative to assess treatment effects. Their use can improve both the efficiency and feasibility of basket trials, particularly in ultra-rare indications where conventional comparators are neither practical nor ethical.

To support the integration and interoperability of these diverse data streams, initiatives such as (ERICA) have been launched. ERICA connects European Reference Networks (ERNs), patient registries, biobanks, and clinical trial platforms, with the goal of harmonizing data collection standards and fostering seamless exchange across research infrastructures. This coordinated ecosystem strengthens the methodological underpinnings of rare disease research and enables a more scalable and inclusive approach to evidence generation.

Looking forward, emerging applications of artificial intelligence—particularly in the development of digital twins or jumeaux numériques—offer further potential to transform trial methodologies. These AI-driven models, which simulate disease progression and individual treatment responses based on multimodal patient data, could serve as dynamic comparators or predictive tools for optimizing trial design and personalization. While still in early stages, the integration of digital twins into basket trial frameworks may ultimately enhance predictive accuracy, reduce trial burden, and facilitate regulatory-grade evidence in rare and heterogeneous populations.

Collectively, these innovations underscore the critical importance of data integration and advanced analytics in overcoming the structural challenges of rare disease basket trials.

#### Decentralized and adaptive methodologies

Adopting hybrid trial designs, including decentralized models with remote monitoring technologies and mobile clinical research teams, can reduce patient burden and expand access to trials [96]. Implementing local specimen collection networks further enhances trial feasibility, particularly in geographically diverse patient populations. Additionally, utilizing Bayesian adaptive designs—statistical frameworks that allow ongoing data to inform pre-specified modifications such as sample size, treatment allocation, or study arms—enables more efficient sample size optimization and increases the likelihood of meaningful results despite smaller participant numbers[97].

In this context, the REALISE-D project—a European public–private partnership project launched under the Innovative Health Initiative (IHI) [28]—aims to develop scalable decentralized trial models specifically tailored for RDs. By leveraging among other methods, digital health tools, telemedicine, and patient-centered data platforms, REALISE-D is working to overcome geographical and logistical barriers that often limit participation in traditional clinical trials. Its outputs are expected to serve as a blueprint for future decentralized and adaptive methodologies in the rare disease space, particularly for complex multi-arm or basket trial designs.

By systematically implementing these strategies, the rare disease research community can transform existing patient recruitment challenges into opportunities for innovation. When combined with advanced methodologies, basket trials have the potential to significantly accelerate therapeutic development for RDs, all while maintaining the scientific rigor required for regulatory approval. Future research should focus on quantifying the impact of these strategies on recruitment rates, trial efficiency, and ultimately, patient outcomes, as this will provide valuable insights into optimizing basket trial designs for rare disease applications.

Taken together, these observations highlight both the promise and the complexity of basket trials in RDs. While basket designs can provide pragmatic value—particularly in exploratory studies, drug repurposing, or trials guided by shared molecular mechanisms—they should not be viewed as a universal solution. Instead, their use must be carefully weighed against feasibility, ethical considerations, and regulatory expectations. Going forward, methodological innovations such as adaptive and Bayesian designs, alongside earlier and more transparent engagement with regulators, will be critical to ensuring that basket trials are applied judiciously and only where they can meaningfully advance therapeutic development for underserved patient populations.

## Limitations

While this review employed a systematic and structured approach to identify and analyze basket trials conducted in the context of RDs, several limitations must be acknowledged. Despite comprehensive database screening and the use of multiple search strategies, it remains possible that certain relevant studies were not captured—particularly unpublished or non-registered trials, or those categorized under broader or evolving terminologies that may obscure their classification as basket trials. Consequently, the review may not be entirely exhaustive in cataloging all relevant initiatives in this area.

Furthermore, the primary objective of this work was to characterize and synthesize the current landscape of basket trials in RDs, with particular emphasis on trial design, methodological innovations, and patient recruitment strategies. This review did not aim to formally assess the methodological quality or risk of bias of each included study, as recommended by PRISMA guidelines, nor did it provide a detailed statistical appraisal of the trial designs or their relative efficacy. These aspects—while essential—fell beyond the scope of this overview, which was conceived as a high-level analysis to inform future directions in trial development, especially in non-oncological RDswhere basket trials remain underutilized.

In addition, the review did not explore in depth the regulatory implications of adaptive trial designs or the role of qualitative natural history data—often essential in RDsand typically shared across expert networks—to inform trial feasibility and endpoint selection. Future work should build upon this initial synthesis by integrating formal quality assessments, comparative outcome analyses, and a deeper investigation of how real-world data, natural history cohorts, and regulatory frameworks shape the design and acceptability of basket trials in rare disease contexts.

Nonetheless, this review offers a timely and comprehensive overview of the evolving role of basket trials in RDs, highlighting both the opportunities and methodological challenges associated with their broader implementation across diverse clinical and regulatory settings.

## Conclusion and next steps

Basket trials represent a promising methodological approach for rare disease research, offering pragmatic solutions to the challenges posed by small, heterogeneous, and geographically dispersed patient populations. Their growing adoption in oncology demonstrates clear advantages in feasibility, efficiency, and regulatory acceptance, particularly through molecularly defined, biomarker-informed designs. However, their application in non-oncological RDs remains limited, constrained by a lack of validated biomarkers, inconsistent endpoints, and regulatory uncertainty.

To unlock their full potential beyond oncology, three critical priorities emerge. First, greater harmonization of outcomes is needed through the development of core outcome sets, integration of patient-reported outcome measures, and the adoption of validated digital biomarkers, ensuring results are both patient-centered and regulatorily acceptable. Second, regulatory clarity and alignment across jurisdictions will be essential to support innovative, adaptive designs and foster confidence in single-arm or small-sample studies. Third, sustained investment and cross-sector collaboration—spanning industry, academia, regulatory bodies, and patient advocacy organizations—are crucial to overcome feasibility barriers and embed patient perspectives at the heart of trial design.

Collaborative frameworks such as DREAMS, INVENTS, and REALISE-D illustrate how Europe is advancing methodological innovation, digital integration, regulatory dialogue and patient-centered approaches. Complementary efforts, including the FDA’s Rare Disease Endpoint Advancement (RDEA) program [98] and FDA–EMA joint initiatives, highlight the importance of extending these advances globally. Leveraging such regional and transatlantic models in a coordinated manner will accelerate endpoint validation and trial harmonization, ensuring that basket trial innovations benefit rare disease populations worldwide.

With sustained innovation and international collaboration, basket trials can evolve from a predominantly oncological tool to a transformative framework for rare disease research more broadly, expanding access and driving therapeutic development for underserved populations.

## Supplementary Information

Supplementary Material 1.

## Data Availability

All data analyzed in this review were obtained from publicly available sources, including Medline, Embase, ClinicalTrials.gov, the EU Clinical Trials Register, WHO-ICTRP, Google Scholar, and the Google search engine. The final dataset of included basket trials is available upon request from the corresponding author.
