# Correction: Basket trials in rare diseases: a systematic review of current practices, methodological challenges, and future directions

**DOI:** 10.1186/s13023-025-04149-6

**Published:** 2026-03-03

**Authors:** Wael Khazen, Solange Corriol-Rohou, Teresinha Evangelista, Arnaud Valent, Sarah Abbas, Xavier Nissan, Alexandre Mejat

**Affiliations:** 1 AFM-Telethon, 1, rue de l’Internationale, BP 59, 91002 Evry cedex, France; 2 SCR Consulting, 75016 Paris, France; 3 Unité de Morphologie Neuromusculaire, Institut de Myologie, Paris, France; 4 Généthon, 1 bis, rue de l’Internationale, 91002 Evry cedex, France; 5 Université Paris-Saclay, Université d’Evry, Inserm, IStem, UMR861, 91100 Corbeil-Essonnes, France; 6 IStem, CECS, 91100 Corbeil-Essonnes, France

**Correction to**: ***Orphanet J Rare Dis*** **20**,** 578 (2025).** 10.1186/s13023-025-04048-w

Following publication of the original article [1], the authors reported an error in the name of the author Arnaud Valent. The name present in this correction is correct.

The original article [1] has been updated.
